# Weight loss from diagnosis of Crohn’s disease to one year post-diagnosis results in earlier surgery

**DOI:** 10.1038/s41598-023-48474-x

**Published:** 2023-11-30

**Authors:** Minjee Kim, Minsung Cho, Sungjun Hong, Joo Hye Song, Eun Ran Kim, Sung Noh Hong, Dong Kyung Chang, Young-Ho Kim, Ji Eun Kim

**Affiliations:** 1 Department of Medicine, Samsung Medical Center, Sungkyunkwan University School of Medicine, 81 Irwon-ro, Gangnam-gu, Seoul, 06351 Republic of Korea; 2 Department of Digital Health, Samsung Advanced Institute for Health Sciences and Technology, Sungkyunkwan University, Seoul, Republic of Korea; 3 Department of Medicine, Konkuk University Medical Center, Seoul, Republic of Korea

**Keywords:** Crohn's disease, Digestive signs and symptoms

## Abstract

Malnutrition might play a key role in the prognosis of patients with Crohn’s disease (CD). The aim of this study was to explore the impact of weight loss from diagnosis of CD to one-year post-diagnosis on disease prognosis in terms of surgery. Patients who were diagnosed with CD at Samsung Medical Center between 1995 to 2020 were included in this study. The study defined the “group with weight loss” as patients with weight loss in one year after diagnosis and the “group without body weight loss” as patients without weight loss in one year after diagnosis. Their data such as demographics, laboratory findings, and medical interventions were collected retrospectively. The primary outcome was confirmation of the difference in the incidence of surgery associated with CD between the group with weight loss and the group without body weight loss. We further analyzed factors associated with surgery outcomes. A total of 165 patients were analyzed in this study. Forty-one patients (24.8%) had body weight loss whereas 124 patients (75.2%) had no body weight loss. Body change at one year showed no significant association with direct surgical incidence. However, the patients with weight loss tended to undergo surgery earlier than patients without body weight loss. Among factors associated with outcomes of Crohn’s surgery, the albumin was the only significant factor. Patients with weight loss had no statistically significant increase in the risk of surgery than patients without weight loss, although they tended to undergo surgery earlier than patients without body weight loss. A prospective study is needed to determine serial body weight changes during follow-up for patients with CD.

## Introduction

Crohn’s disease (CD) is a transmural inflammation condition that may lead to fibrosis, strictures, and obstruction. It can also lead to microperforation and fistula formation^1^. In order to prevent from surgery, it is necessary to predict the risk of surgery and to treat it beforehand. CD can impair nutrition status, leading to bone disease, and nutrition deficiencies^2^. It is crucial to easily measure and confirm nutritional status to prevent surgery in CD patients.

Protein and albumin levels commonly indicate nutrition levels. It is well known that albumin is a significant factor affecting surgery in patients with CD^3^. A previous study has demonstrated that the prevalence of sarcopenia is high in patients with CD requiring bowel resection. One study showed that a sarcopenia group had longer operation time and greater hemorrhage volume than the non-sarcopenia group^4^. In addition, sarcopenia can significantly increase the risk of major postoperative complications^5^.

The most common micronutrient deficiencies in Inflammatory bowel disease (IBD) patients concern iron, calcium, selenium, zinc, magnesium, water-soluble vitamins, in particular, B12 and folic acid, and fat-soluble vitamins, such as A,D, and K^6,7^. Anemia is the most frequency systemic complication and low bone mass and osteoporosis are common in IBD. In addition, folic acid deficiency may lead to hyperhomocysteinemia, which is an established risk factor for many cardiovascular disease and increased incidence of arterial and venous thromboembolic events in CD^8^. Folate deficiency is also known as an established risk factor of colorectal cancer in the IBD population^9^. Vitamin B12 deficiency is associated with megaloblastic anemia and peripheral neuropathy, and hyperhomocysteineia, an independent risk factor for thromboembolism^10^. In addition, as food consumption is one of the cultural and social activities, psychosocial roles may be altered in patients with IBD^11^.

To treat IBD patients, compliance must be well-checked and patients must be able to understand the physician’s medical decision. However, sarcopenia cannot be used as a practical clinical indicator. Analysis of different quick parameters of malnutrition in one study has revealed that Body Mass Index (BMI) and serum albumin have high predictive values in IBD patients with active disease^12^. The BMI as the quick static parameter is simple and convenient method for routine assessment of nutritional status in patients with active IBD^12^. However, the results of previous studies on BMI nutritional parameters are inconsistent^13–19^.

There is no study on dynamic indicators for patients with CD. No previous study has evaluated the association of weight loss of adult patients with CD and surgical outcomes. Thus, the aim of this study was to explore the impact of weight loss from diagnosis of CD to one year after diagnosis on surgery. We aimed to develop a quick indicator that could be easily practiced in daily life for patients with CD.

## Materials and methods

### Study design, setting, and participants

**Figure 1:**
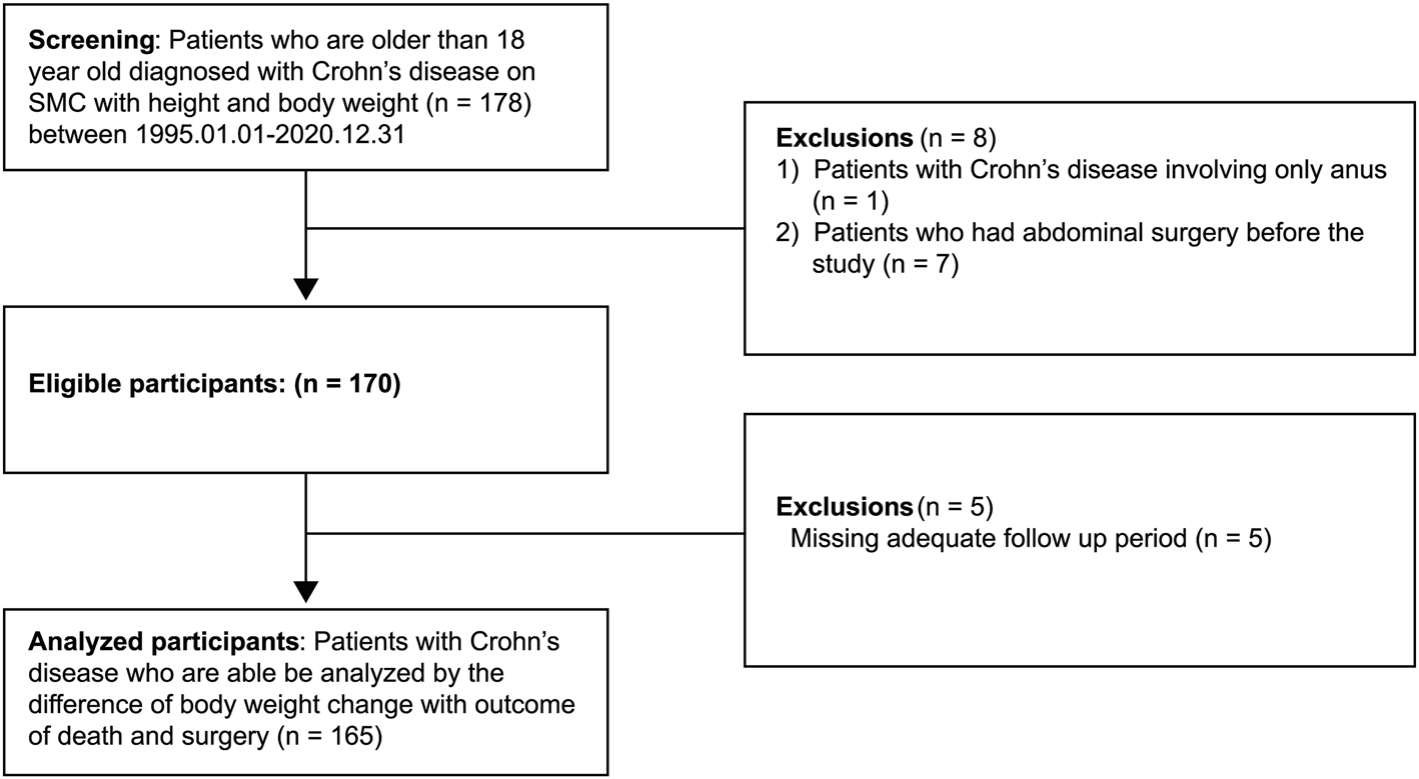
Study population. *SMC* Samsung Medical Center.

This was a retrospective cohort study conducted at Samsung Medical Center (SMC), a tertiary academic institution in Seoul, South Korea. A total of 178 patients older than 18 years who were diagnosed with CD with height and body weight recorded in electronic medical record in the gastroenterology outpatient clinic of SMC from January 1995 to December 2020 were reviewed. Exclusion criteria were patients with CD involving only the anus (n = 1), those who had abdominal surgery before the study (n = 7), and those who did not have an adequate follow-up period (n = 5). Finally, a total of 165 patients diagnosed with CD were included and analyzed (Fig. 1).

We divided patients into two groups. We defined the “group with weight loss” as patients who had weight loss in one year after diagnosis and the “group without body weight loss” as patients without weight loss in one year after diagnosis. A total of 41 patients (24.8%) had body weight loss and 124 patients (75.2%) had no body weight loss from the time of the first diagnosis. This study compared age, sex, status of smoking, BMI, and laboratory findings such as hemoglobin (Hb), albumin, white blood cell (WBC), platelets, C-reactive protein (CRP), and erythrocyte sedimentation rate (ESR) at diagnosed between the two groups. According to World Health Organization(WHO) criteria of BMI, subjects were categorized as underweight (BMI < 18.5 kg/m^2^), normal (18.5 kg/m^2^ ≤ BMI ≤ 24.9 kg/m^2^), overweight (25.0 kg/m^2^ ≤ BMI ≤ 29.9 kg/m^2^), and obese (BMI ≥ 30 kg/m^2^). According to Asian criteria of BMI, subjects were classified as underweight (BMI < 18.5 kg/m^2^), normal (18.5 kg/m^2^ ≤ BMI ≤ 23.0 kg/m^2^), overweight (23.0 kg/m^2^ < BMI ≤ 27.5 kg/m^2^), and obese (BMI > 27.5 kg/m^2^). In addition, in the follow-up period, disease location, perianal disease, death, surgery, extra-gastrointestinal (GI) symptoms (peripheral arthritis, ankylosing spondylitis, erythema nodosum, pyoderma gangrenosum, primary sclerosing cholangitis) and drug history (immune modulators as mercaptopurine, azathioprine, methotrexate; biological agents as anti-tumor necrosis factor (Anti-TNF), adalimumab, ustekinumab, vedolizumab, golimumab, certolizumab) were evaluated.

We reviewed Samsung medical center’s electronic medical record (EMR), which included longitudinal information such as medical and pharmaceutical records, disease diagnoses, and prescribed medications. This study was approved by the Institutional Review Board (IRB) of Samsung Medical Center (IRB file number: Samsung Medical Center 2022-08-032). It was conducted under the Declaration of Helsinki. An exemption from informed consent was granted by the IRB of “Samsung Medical Center” because all data were analyzed anonymously.

### Variables and definition

During check-ups, surveys on medical history and laboratory tests were performed. We collected patient’s age, sex, height, body weight, diagnosis date, and smoking when diagnosed. Patients’ height and body weight were collected a year later after the diagnosis date. Blood pressure (BP) was measured by autonomic sphygmomanometers on the patient’s right arm after at least 5 min of rest. BMI was defined as body weight in kilograms divided by height in meters squared (kg/m^2^). Medical history was evaluated thoroughly for disease location with CD involvement including anus (anal fistula), extra gastrointestinal symptoms (such as peripheral arthritis, ankylosing spondylitis, erythema nodosum, pyoderma gangrenosum, and primary sclerosing cholangitis), surgery associated with CD, medication, and death throughout the follow-up period. The perianal disease was defined as perianal fistula, perianal abscess, and anal canal lesions such as anal fissures and anal strictures. Drug history was classified as either immune modulators or biologic agents. Immune modulators such as mercaptopurine, azathioprine, and methotrexate and biologic agents such as anti-tumor necrosis factor (anti-TNF), adalimumab, ustekinumab, vedolizumab, golimumab, and certolizumab were evaluated. Laboratory findings such as hemoglobin (Hb), albumin, white blood cell, platelets, C-reactive protein (CRP), and erythrocyte sedimentation rate (ESR) were compared. Surgery associated with CD was defined as bowel resection for the part involved in CD (including anal fistula) from January 1995 to December 2020.

The primary outcome was confirmation of the difference in the incidence of surgery associated with CD between the group with weight loss and the group without body weight loss. We further analyzed factors associated with surgery outcomes.

### Statistical analysis

For comparison between the two groups, independent samples t-test or Mann–Whitney U-test according to Shapiro–Wilk test for normality was used for continuous variables and chi-square test was used for categorical variables. Values are expressed as median (interquartile range) for continuous variables and number (%) for categorical variables. A 1:1 propensity-score (PS) matching was done by 1:1 greedy matching with a caliper width of 0.2 times the pooled SD of the logit without replacement. This study evaluated variables of standardized mean difference (SMD) of 0.2 + age and perianal disease for baseline characteristics.

In order to assess associations of covariates with the outcome, a logistic regression model was used. The cumulative incidence of surgery was estimated using a density plot and the Kaplan–Meier method. All tests were two-sided with a statistical significance level of p-value < 0.05. All statistical analyses were executed using R Statistical Software (version 4.1.0; R Foundation for Statistical Computing, Vienna, Austria).

## Results

### Baseline characteristics

The baseline clinical characteristics of study participants according to the two groups are summarized in Table 1. Of a total of 165 patients, the median age when diagnosed was 29 (20–41) years. There were 121 (73.3%) male patients. A total of 7 (4.2%) patients died in the study period and 35 (21.2%) patients underwent surgery. The two groups showed no significant difference in sex, BMI, disease location, hemoglobin, albumin, white blood cell, platelets, CRP, ESR, death, surgery, extra GI symptoms, or medications. The only significant difference between the two groups was smoking (p-value < 0.05). After propensity matching, the average albumin level was 4.1 (0.50). The total number of 18 (26.5%) underwent surgery. A total of 47 (69.5%) patients had a normal BMI based on WHO BMI criteria and 38 (55.9%) patients had a normal BMI based on Asian criteria. Similarly, a total of 16 (23.5%) patients had a underweight BMI according to both WHO and Asian criteria. They were evenly categorized to two groups of body weight loss and non-body weight loss.

**Table 1:** Baseline characteristics with propensity matching between two groups.

Group	Before PS matching			P value	After PS matching			SMD
	Total	Bwt loss ( +)	Bwt loss (-)		Total (n = 68)	Bwt loss ( +) (n = 34)	Bwt loss (-) (n = 34)	
	(n = 165)	(n = 41)	(n = 124)					
Age (median [IQR])	29.00 [20.00, 41.00]	30.00 [23.00, 39.00]	29.00 [19.00, 41.00]	0.35	32.82 (14.19)	31.26 (14.05)	34.38 (14.37)	0.21
Sex (%)				0.07				0.14
Female	44 (26.7)	6 (14.6)	38 (30.6)		14 (20.6)	6 (17.6)	8 (23.5)	
Male	121 (73.3)	35 (85.4)	86 (69.4)		54 (79.4)	28 (82.4)	26 (76.5)	
Smoking (%)				0.02				0.31
Non-smoker	137 (83.0)	31 (75.6)	106 (85.5)		56 (82.4)	30 (88.2)	26 (76.5)	
Smoker	12 (7.3)	7 (17.1)	5 (4.0)		6 (8.8)	2 (5.9)	4 (11.8)	
Ex-smoker	16 (9.7)	3 (7.3)	13 (10.5)		6 (8.8)	2 (5.9)	4 (11.8)	
BMI_WHO (%)				0.24				0.24
Underweight	47 (28.5)	9 (22.0)	38 (30.6)		16 (23.5)	8 (23.5)	8 (23.5)	
Normal	99 (60.0)	27 (65.9)	72 (58.1)		47 (69.1)	23 (67.6)	24 (70.6)	
Overweight	18 (10.9)	4 (9.8)	14 (11.3)		4 (5.9)	2 (5.9)	2 (5.9)	
Obese	1 (0.6)	1 (2.4)	0 (0.0)		1 (1.5)	1 (2.9)	0 (0.0)	
BMI_Asian (%)				0.58				< 0.01
Underweight	47 (28.5)	9 (22.0)	38 (30.6)		16 (23.5)	8 (23.5)	8 (23.5)	
Normal	83 (50.3)	23 (56.1)	60 (48.4)		38 (55.9)	19 (55.9)	19 (55.9)	
Overweight	27 (16.4)	6 (14.6)	21 (16.9)		12 (17.6)	6 (17.6)	6 (17.6)	
Obese	8 (4.8)	3 (7.3)	5 (4.0)		2 (2.9)	1 (2.9)	1 (2.9)	
Disease location (%)				0.30				0.06
Ileum	49 (29.7)	11 (26.8)	38 (30.6)		17 (25.0)	9 (26.5)	8 (23.5)	
Colon	12 (7.3)	1 (2.4)	11 (8.9)		2 (2.9)	1 (2.9)	1 (2.9)	
Ileocolon	104 (63.0)	29 (70.7)	75 (60.5)		49 (72.1)	24 (70.6)	25 (73.5)	
Perianal.disease (%)				0.55				< 0.01
0 (None)	92 (55.8)	25 (61.0)	67 (54.0)		38 (55.9)	19 (55.9)	19 (55.9)	
1 (Present)	73 (44.2)	16 (39.0)	57 (46.0)		30 (44.1)	15 (44.1)	15 (44.1)	
Hb (mean (SD))	12.6 (2.06)	13.1 (1.95)	12.4 (2.08)	0.07	12.74(1.94)	12.8 (1.97)	12.7 (1.94)	0.08
Albumin (median [IQR])	4.1 [3.70, 4.40]	4.2 [3.80, 4.50]	4.1 [3.68, 4.40]	0.34	4.1 (0.50)	4.1 (0.51)	4.1 (0.50)	0.03
WBC (median [IQR])	7.0 [5.35, 9.05]	7.3 [5.60, 9.19]	6.85 [5.30, 8.85]	0.35	7.86 (3.62)	7.77 (4.24)	7.95 (2.92)	0.05
Platelets (median [IQR])	303.00 [234.00, 381.00]	315.00 [232.00, 361.00]	301.00 [236.25, 389.00]	0.84	315.31 (108.47)	325.06 (121.33)	305.56 (94.72)	0.18
CRP (median [IQR])	0.77 [0.14, 2.69]	0.77 [0.07, 2.24]	0.76 [0.24, 2.95]	0.22	0.62 [0.11, 2.14]	0.92 [0.08, 2.39]	0.45 [0.12, 1.09]	0.14
ESR (median [IQR])	25.00 [12.00, 51.00]	33.00 [10.00, 52.00]	23.50 [12.75, 50.25]	0.81	36.43 (28.4)	37.79 (27.4)	35.06 (29.8)	0.09
Extra GI Symptoms (%)				0.15				0.26
0 (No)	121 (73.3)	26 (63.4)	95 (76.6)		48 (70.6)	22 (64.7)	26 (76.5)	
1 (Yes)	44 (26.7)	15 (36.6)	29 (23.4)		20 (29.4)	12 (35.3)	8 (23.5)	
Biologics (%)				0.90				0.34
0 (No)	45 (27.3)	12 (29.3)	33 (26.6)		17 (25.0)	6 (17.6)	11 (32.4)	
1 (Yes)	120 (72.7)	29 (70.7)	91 (73.4)		51 (75.0)	28 (82.4)	23 (67.6)	
Immunomodulator (%)				0.97				0.30
0 (No)	62 (37.6)	16 (39.0)	46 (37.1)		25 (36.8)	10 (29.4)	15 (44.1)	
1 (Yes)	103 (62.4)	25 (61.0)	78 (62.9)		43 (63.2)	24 (70.6)	19 (55.9)	
Death (%)				0.27				0.35
0 (No)	158 (95.8)	41 (100.0)	117 (94.4)		66 (97.1)	34 (100.0)	32 (94.1)	
1 (Yes)	7 (4.2)	0 (0.0)	7 (5.6)		2 (2.9)	0 (0.0)	2 (5.9)	
Outcome (%)				0.43				< 0.01
0 (No)	130 (78.8)	30 (73.2)	100 (80.6)		50 (73.5)	25 (73.5)	25 (73.5)	
1 (Yes)	35 (21.2)	11 (26.8)	24 (19.4)		18 (26.5)	9 (26.5)	9 (26.5)	

*Bwt* Body weight, *BMI* Body mass index, *Hb* Hemoglobin, *WBC* White blood cell, *CRP* C-reactive protein, *ESR* Erythrocyte Sedimentation Rate.

### Risk factors of surgical outcome

This study analyzed a total of 165 patients with a logistic regression model between two groups based on surgical outcomes (Table 2). In univariable analysis, patients without body weight loss group were less likely to undergo surgery than patients with body weight loss (Odds ratio (OR): 0.66; 95% CI 0.292–1.530, p = 0.31). Risks of surgery increased in ex-smokers (OR: 1.42, 95% CI 0.375–4.470, p = 0.57) and current smokers (OR: 3.05, 95% CI 0.845–10.332, p = 0.07) than in non-smokers. Based on WHO and Asian BMI criteria, underweight patients were more likely to undergo surgery than those with a normal BMI (OR: 2.73, 95% CI 1.23–6.081, p = 0.01 and OR 2.57, 95% CI 1.138–5.880, p = 0.02 respectively.) Since there were no obese patients in the study, odds ratio was infinite when calculated. Compared to the group with CD involving the ileum, the group with CD involving the ileo-colon had a higher chance of undergoing surgery (OR: 2.64, 95% CI 1.073–7.507, p = 0.05). Patients with a perianal disease were more likely to undergo surgery than those without a perianal disease (OR: 1.25, 95% CI 0.587–2.646, p = 0.56). Immunomodulator users were also more like to undergo surgery than non- immunomodulator users (OR: 2.93, 95% CI 1.252–7.736, p = 0.02).

**Table 2:** Factors associated with outcomes of Crohn’s surgery (N = 165).

	Univariate analysis		Multivariate analysis	
	OR [95% CI]	p-value	OR [95% CI]	p-value
Bwt (Loss(+) vs Loss(−))	0.66 [0.292, 1.530]	0.31	0.68 [0.265, 1.789]	0.43
Age	0.98 [0.957, 1.010]	0.25		
Sex (F vs M)	0.74 [0.333, 1.726]	0.47		
Smoking (Non-smoker)		0.19		
Smoker	3.04 [0.845, 10.332]	0.07		
Ex-smoker	1.42 [0.375, 4.470]	0.57		
BMI_WHO (Normal)		0.03		
Underweight	2.73 [1.238, 6.081]	0.01		
Overweight	0.28 [0.015, 1.535]	0.23		
Obese	0.00 [NA, infinity]	0.99		
BMI_Asian (Normal)		0.06		0.31
Underweight	2.57 [1.138, 5.880]	0.02	1.91 [0.735, 5.023]	0.18
Overweight	0.57 [0.124, 1.906]	0.40	0.48 [0.097, 1.791]	0.31
Obese	0.00 [NA, infinity]	0.99	0 [NA, infinity]	0.99
Disease location (Ileum)		0.07		0.17
Colon	0.65 [0.033, 4.393]	0.70	0.51 [0.024, 3.888]	0.57
Ileocolon	2.64 [1.073, 7.507]	0.05	2.28 [0.802, 7.334]	0.14
Perianal disease (None vs Present)	1.25 [0.587, 2.646]	0.56		
Hb	0.88 [0.732, 1.052]	0.16	1.22 [0.906, 1.666]	0.21
Albumin	0.37 [0.196, 0.681]	< 0.01	0.40 [0.163, 0.917]	0.03
WBC	0.98 [0.858, 1.104]	0.75		
Platelets	1.00 [0.999, 1.005]	0.17	1.00 [0.996, 1.004]	0.94
CRP	1.14 [0.980, 1.328]	0.08	0.94 [0.750, 1.172]	0.61
ESR	1.01 [1.002, 1.027]	0.03	1.01 [0.989, 1.030]	0.36
Extra GI Symptoms (No vs Yes)	1.89 [0.837, 4.154]	0.12	1.99 [0.744, 5.243]	0.16
Biologics (No vs Yes)	1.34 [0.579, 3.406]	0.51		
Immunomodulator (No vs Yes)	2.93 [1.252, 7.736]	0.02	2.24 [0.863, 6.435]	0.11

*Bwt* Body weight, *BMI* Body mass index, *Hb* Hemoglobin, *WBC* White blood cell, *CRP* C-reactive protein, *ESR* Erythrocyte Sedimentation Rat.

In multivariate logistic regression, underweight patients were more likely to undergo surgery than patients with normal body weight (OR: 1.91, 95% CI 0.735–5.023, p = 0.18). Overweight patients underwent surgery with OR of 0.48 (95% CI 0.097–1.791, p = 0.30). The obese patients’ odds ratio was unmeasurable due to the small number of obese patients in this study. BMI and location of colon involvement were not significant factors of CD. Hemoglobin (OR: 1.22, 95% CI 0.906–1.666, p = 0.21) and ESR (OR: 1.01, 95% CI 0.989–1.030, p = 0.36) tended to increase the risk of surgery whereas CRP tended to decrease the risk of surgery (OR: 0.94, 95% CI 0.750–1.172, p = 0.61). Albumin is a statistically significant factor in the risk of surgery (OR: 0.40, 95% CI 0.163–0.917, p = 0.03).

### Development of surgery

**Figure 2:**
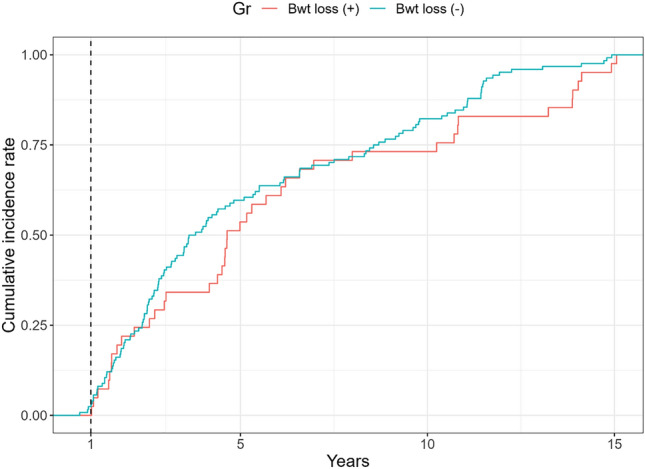
Cumulative Incidence of surgery by density plot. *Bwt* Body weight.

The density plot shows the cumulative incidence of surgery during the follow-up duration (Fig. 2). It shows data from the date of diagnosis to the date of surgery for patients who underwent surgery or the last outpatient visits date for patients who did not undergo surgery. The frequency of surgery was compared between the group with body weight loss and the group without body weight loss. Outpatient clinic visit and surgery rates showed a relatively increasing pattern in patients who had body weight loss than in patients who had no body weight loss at one year after CD diagnosis. This trend continued five years after CD diagnosis. The incidence of surgery was also evaluated by Kaplan–Meier curve (Supplementary Fig. S1). At 15 years, there was one patient in the body weight loss group and 0 patient in the non-body weight loss group. More patients were censored in the first five years in the non-body weight loss group. There was no difference in the time to the event between the two groups.

## Discussion

In this study, we tried to develop a simple indicator for surgery of patients with CD by body weight loss during one year from the time of diagnosis. Body weight loss during one year tended to increase surgery, although it was not a significant factor statistically. In addition, the patients with body weight loss tended to undergo surgery earlier than those without weight loss. The albumin was the only statistically significant factor associated with surgical outcome. In the group with body weight loss, the incidence of surgery suddenly increased at around the first one year through density plot analysis. To the best of our knowledge, this is the first study to demonstrate the association between body weight differences in the first year after diagnosis and the ultimate surgical outcome in patients with CD.

The body weight loss group had a significantly higher proportion of smokers than the body weight gain or no change group. In addition, smoker increased surgery with odds ratio of 3.04 compared to non-smoker and ex-smoker increased surgery with odds ratio of 1.42 in our study. Smoking is associated with an increased risk of CD. It is well known that smoking can increase the risk of complications from CD (e.g., strictures, fistula) and the need for surgery^20–22^. The smoking may alter the intestinal microbiota ^23^, and it is associated with an altered immune response, with abnormal cascade of inflammation in the intestinal mucosa^24^. As smoking is associated with inflammation of the intestine, it leads to weight loss in CD. The benefits of smoking cessation are considerable; after cessation, no difference in surgical CD recurrence or postoperative acute CD relapses exists between ex-smokers and nonsmokers^25^. Even though definite causal relation between smoking, weight loss and CD is unknown, smoking cessation should be advised in patients with CD.

Malnutrition might play a key role in the prognosis of patients with CD. Several previous studies have proven that BMI is one of the most predictive parameters to assess the nutritional status of IBD patients^12,22^. One study has also investigated specific nutritional deficiencies with nutrition assessment for IBD patients and found that BMI is not a factor affecting disease activity^19^. Some studies have discovered that BMI was lower in patients with IBD than in controls without IBD^12,26–28^. Some other studies have shown a strong independent negative impact of malnutrition on CD^29,30^. Perioperative nutritional status has an important effect on post-operative conditions and recurrence in people with active CD^31,32^. While surgery often leads to clinical remission of CD, most patients eventually relapse. The risk and severity of recurrence after surgical resection are variable. Therefore the patients with CD need to be balanced with preventive medical therapy^33^. As dietary intake is another important factor influencing nutritional status, clinicians should consider more concise dietary supplement to patients with CD.

It is well known that albumin is a significant factor affecting surgery in patients with CD^3^. However, as serum albumin is one of the active phase reactants, the serum albumin itself cannot describe the nutrition status as a whole. Previous studies show that low albumin levels are the results of the combined effects of inflammation and inadequate caloric intake^34–36^. As hypoalbuminemia is independently associated with both malnutrition and inflammation in patients with CD and was most profound in subjects with both malnutrition and active inflammation, these results suggest that low serum albumin may be a marker of either malnutrition or active disease^35^. However, as median CRP, one of the robust inflammatory markers, is normal in the study, researchers can rely on albumin level to a certain extent.

There seems no uniform consensus about the prognosis of CD in terms of disease location. A study of 280 patients in John Radcliffe Hospital (Oxford, UK) concluded that patients with ileal disease had significantly higher postoperative recurrence rates than patients with ileocolonic (Montreal-L2) or colonic disease (Montreal-L3)^37^. On the contrary, some studies have shown no correlation between disease location and prognosis^38,39^. In our study, the numbers of patients with ileum, colon, and ileo-colon involved CD were 49, 12, and 104, respectively. These small numbers of patients might be the reason why disease location was significant in univariate analysis but insignificant in multivariate analysis. Results might be different if there were more patients in the study. Managing nutrition in CD patients can be part of the treatment when first diagnosed with CD. There are diverse evaluations of nutritional status in patients with IBD. Duration of disease, activity, and small bowel involvement were considered to be the most influencing factors for nutritional status^13,16,19,40,41^. In our study, there was no difference in the field of small bowel involvement or the duration of disease between weight loss and no weight loss groups. Other well-known nutrition indicators are vitamin levels and related disorder as anemia, bone disease, hypercoagulability, and wound healing^6^. In order to perform these tests, blood sampling is required and procedures that need to be interpreted are required. Thus, it is complicated to confirm with quick indicators.

This study has several limitations. First, as it was a retrospective single-center study, data collection was not complete and bias was inevitable. Despite limited information, we thoroughly reviewed all data related to variables by EMR. Second, this study classified patients into two groups based on body weight alone to find a simple and quick parameter. Body weight solely does not represent sarcopenia. It might not be a nutrition indicator. However, patients with CD can easily self-check their body weights anytime anywhere. A previous study has shown that sarcopenia can adversely outcomes of IBD patients and that sarcopenia can be defined as an L3 skeletal muscle index (SMI). However, to measure sarcopenia, we need an assistant for a radiologist and computer tomography (CT)^42^. Sarcopenia is by no means of a practical indicator. We simply classified weight loss and non-weight loss groups during a fixed period. When patients are categorized by “meaningful weight loss”, results might be different. In baseline characteristics, there was a significant difference in the smoking portion between the two groups, which might have affected the surgical outcome. This study showed a trend in surgical outcome of weight loss and non-weight loss groups. If data on serial body weights are collected, the results might be different. Third, as discussed above, as albumin is one of the active phase reactants, there is limitation explaining hypoalbuminemia as the sole indicator of malnutrition. However, as median CRP is normal in the baseline characteristics, this limitation can be offset to a certain extent. Fourth as there is a sample size difference in body weight loss group and non-body weight loss group, there may be selection bias. However, we addressed this issue by adjusting patient characteristics through the PS matching method.

In summary, nutrition is very important for the prognosis of CD. This study showed that patients with weight loss tended to undergo surgery earlier than patients without body weight loss, although the effect of weight loss at one year after the first diagnosis had no statistically significant effect on the prognosis of CD. Therefore, once a diagnosis of CD is made, not only the appropriate pharmacologic therapy is important but also patients should be encouraged to adhere to a strict diet and nutritional education to improve long-term disease outcomes. Statistically significant large-scale prospective studies or Randomized controlled trial (RCT)s will be required in the future.

### Supplementary Information

Supplementary Information 1.

## Data Availability

All data generated or analyzed during this study are included in this published article (and its Supplementary Information files).

## Abbreviations

CD: Crohn’s disease
BMI: Body mass index
IBD: Inflammatory bowel disease
Hb: Hemoglobin
WBC: White blood cell
CRP: C-reactive protein
ESR: Erythrocyte sedimentation rate
WHO: World Health Organization
GI: Gastrointestinal
Anti-TNF: Anti-tumor necrosis factor
EMR: Electronic medical record
BP: Blood pressure
PS: Propensity-score
SMD: Standardized mean difference
OR: Odds ratio
SMI: Skeletal muscle index
CT: Computed tomography
RCT: Randomized controlled trial
