# Supplementary figures and images for: Weight loss from diagnosis of Crohn’s disease to one year post-diagnosis results in earlier surgery
Source: Sci Rep. 2023 Nov 30;13:21101. doi: 10.1038/s41598-023-48474-x (PMC10689484; doi:10.1038/s41598-023-48474-x)

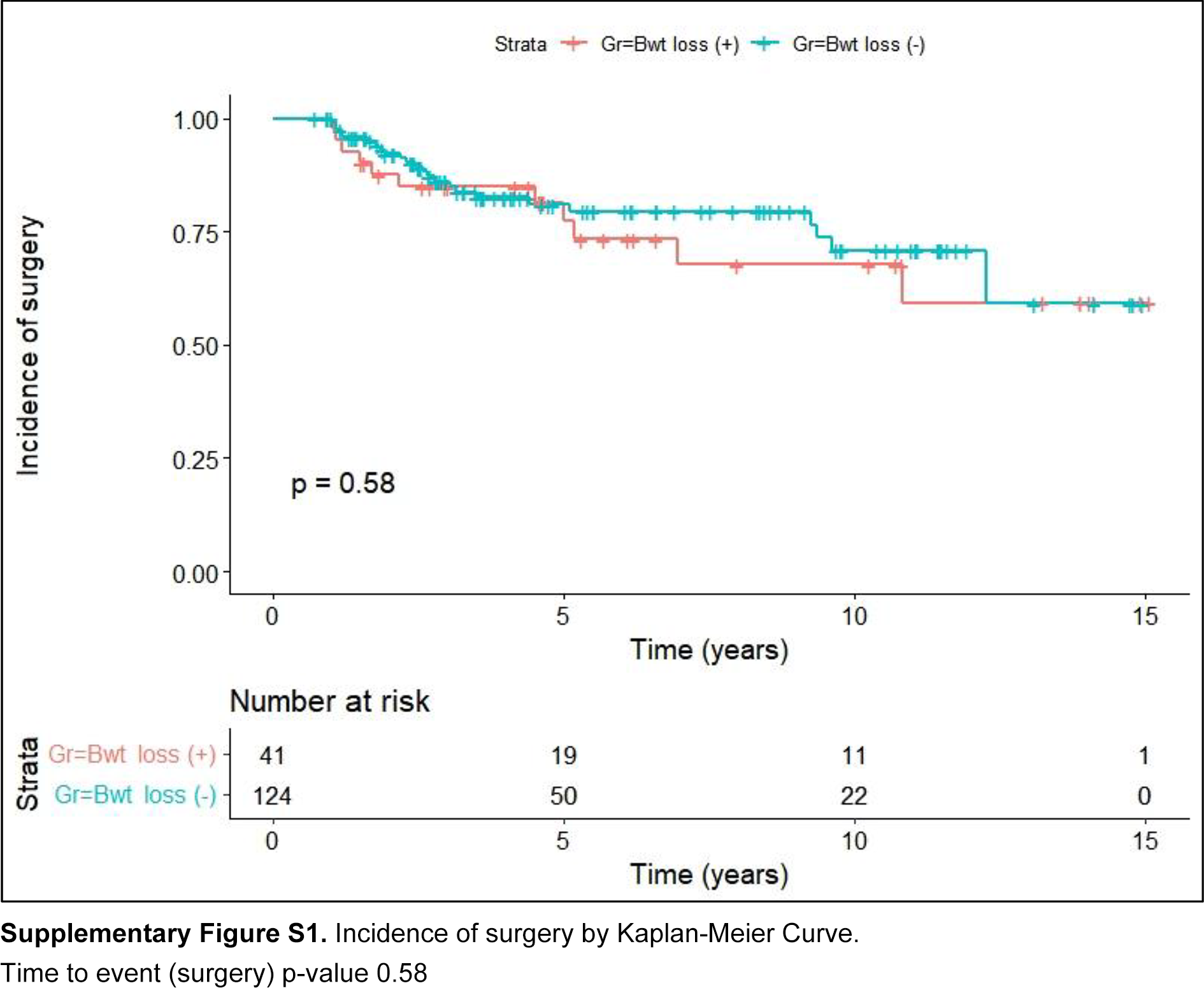

Supplement: Supplementary file 1 — Supplementary Information 1. [file 41598_2023_48474_MOESM1_ESM.tif]
